# Coronary Vasospasm Associated With Synthetic Marijuana Resulting in Transient Electrocardiogram Changes and Troponin Elevation

**DOI:** 10.7759/cureus.25084

**Published:** 2022-05-17

**Authors:** Syed S Fatmi, Andrew Outlaw

**Affiliations:** 1 Internal Medicine, Southeast Health Medical Center, Dothan, USA; 2 Emergency Medicine, Southeast Health Medical Center, Dothan, USA

**Keywords:** myocardial infarction, marijuana, chest pain, acute coronary syndrome, transient, ekg, st elevation, vasospasm

## Abstract

Chest pain is a common presentation in the emergency department requiring prompt evaluation and risk stratification to assess for acute coronary syndrome versus non-cardiac causes. In the past, cocaine has been documented to be associated with the presentation of acute myocardial infarction. This case report describes a rare case of coronary vasospasm with transient electrocardiogram (EKG) changes and significantly elevated troponin in an individual presenting with a recent history of synthetic marijuana use. This report highlights the initial presentation, progression, and management of synthetic marijuana-associated coronary vasospasm mimicking the presentation of acute myocardial infarction. This is a unique case that describes not only the clinical presentation with dynamic EKG changes but also significantly elevated biomarkers, as would be seen with acute myocardial infarction. Challenges associated with synthetic marijuana-mediated coronary vasospasm are not only related to the management but also present a diagnostic challenge because it is not easily detected by urinary drug screen compared to traditional marijuana.

## Introduction

Marijuana is a readily available drug worldwide that comes with its associated adverse long-term effects on overall health but can also cause acute cardiovascular adverse effects. The National Institute of Drug Abuse has declared marijuana as “the most commonly used illicit drug in the US and the third most common cause of drug-related emergency department visits between 2004-2011,” with the presentation of stroke, myocardial infarction, and other conditions [1]. Moreover, the National Health and Nutrition Examination Survey from 2016 estimated two million adults with cardiovascular disease in the United States currently using marijuana or with a history of use in the past [2].

Marijuana is a derivative of *Cannabis sativa* that contains psychoactive components such as 9-tetrahydrocannabinol (THC), which binds to cannabinoid 1 and 2 (CB1 and CB2) receptors [3]. CB1 is expressed in the tissues of the brain, cardiac muscle, liver, gastrointestinal tract, and vascular endothelium [4], whereas CB2 receptors are present in immune cells [5]. Acutely, THC is known to cause an increase in blood pressure and heart rate [6], particularly in a dose-dependent fashion [7], while an increase in the frequency of marijuana use is said to increase the risk of cardiac arrhythmias, such as atrial fibrillation, and myocardial infarction via multiple mechanisms including increased myocardial demand [8,9]. If we evaluate the pathophysiology, marijuana use has been known to cause various adverse cardiovascular side effects including but not limited to increased heart rate and blood pressure, vascular tone, and decreased coronary perfusion secondary to increased vascular tone [1]. With chronic use, there is also an increased risk associated with angina due to vasoconstriction of the central and peripheral system, increase in serum aldosterone, activation of the renin-angiotensin system, secondary hypertension, and diminished upregulation of the parasympathetic nervous system [10]. Studies have also shown evidence for vasculature complications including thrombosis and vasospasm associated with marijuana use [11]. A study from 2017 discussed the association of cardiovascular mortality with increased use of marijuana, which increased the risk of hypertensive crisis resulting in an increased cardiovascular-related mortality burden [12].

## Case presentation

A 35-year-old male with a medical history of hypertension, diabetes, and polysubstance abuse presented to the emergency department (ED) with complaints of severe chest pain for approximately three hours after consuming synthetic marijuana. The patient reported having similar pain in the past after consumption of synthetic marijuana. He reported his pain to be severe, substernal, without any radiation, and associated with nausea. He denied any lightheadedness, palpitations, or syncopal episodes with the commencement of his symptoms. The patient was also hypertensive at the time of presentation, without any other abnormalities noted in vital signs.

**Figure 1:**
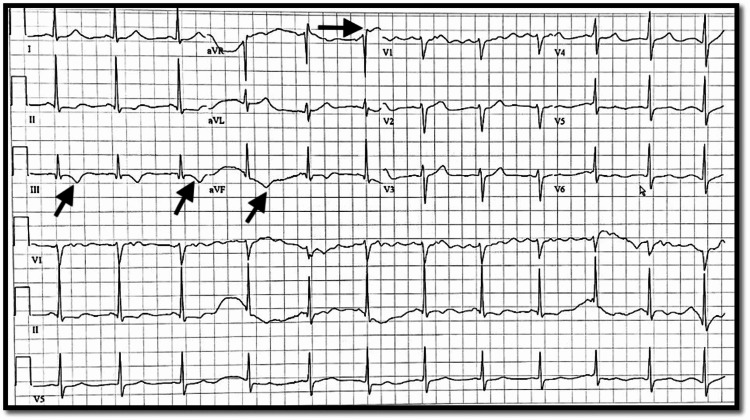
EKG showing ST elevation. Initial EKG showing isolated ST elevation pointed by the horizontal arrow and T-wave inversions in multiple leads indicated by the diagonal arrows. EKG: electrocardiogram

**Figure 2:**
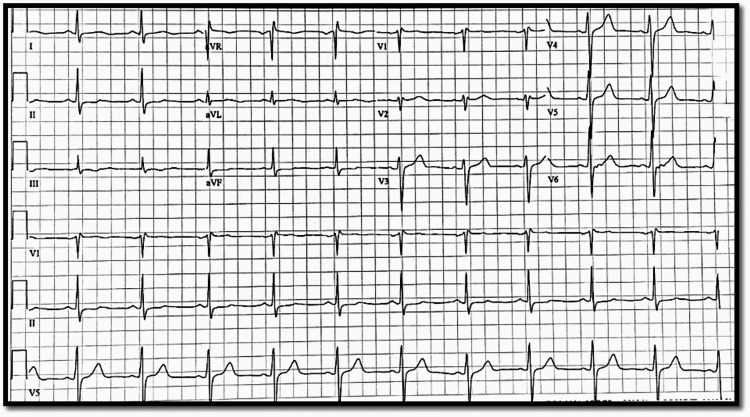
Repeat EKG. No ST elevation noted or T-wave inversion in III aVF or other leads. EKG: electrocardiogram

Initial electrocardiogram (EKG) showed isolated ST elevation from baseline without meeting the criteria of ST-elevation myocardial infarction, associated with T-wave inversions in multiple leads (Figure 1), which were not noted on previous EKGs from a year ago when the patient presented with a similar complaint. Initial troponin was found to be 30.65 ng/mL (reference range <0.04 mg/mL), with no other electrolyte abnormalities noted on the complete metabolic panel. However, the patient was noted to have a leukocytosis with a white blood cell count of 19.8, which was most likely reactive as the patient was afebrile and did not have any other signs or symptoms of infectious etiology. Troponins were trended and continued to rise with a peak of 42.39 mg/mL. The bedside echocardiogram (Echo) was negative for any aortic root dilation, and the chest X-ray (CXR) did not show any mediastinal widening. D-dimer was also ordered which was within the reference range. The patient was started on a heparin and nitroglycerin (nitro) drip, administered aspirin, and cardiology was consulted for further evaluation. Repeat EKG after starting nitro drip did not show a repeat of initial T-wave inversions or isolated ST change from baseline (Figure 2), which was noted at the time of presentation on initial EKG.

Based on the presentation and clinical findings, the patient was taken for a left heart catheterization, which showed diffusely atherosclerotic and irregular coronary arteries with 30% proximal left anterior descending coronary artery stenosis, diffusely irregular right coronary artery up to 40% in mid-segment, irregularities distally up to 10-20%, and angiographic evidence suggesting superficial plaque ulceration involving the mid and distal right coronary artery. The left ventricle was within the upper limits of normal size with an isolated segment of apical inferior severe hypokinesis to akinesis. The patient was maximized on medical management including dual antiplatelet therapy because no single lesion was identified that would be amendable to intervention.

The subsequent Echo, one day after the cardiac catheterization, showed mild concentric left ventricle hypertrophy, low normal global left ventricular systolic function, ejection fraction greater than equal to 50-55%, and no discrete wall motion abnormalities identified in reference to non-ST-elevation myocardial infarction (NSTEMI) that would correlate with such significant troponin elevation. With no significant valvular abnormalities identified, concerns for severe apical hypokinesis were noted on initial catheterization secondary to the vasospastic phenomenon, as the patient did not report any episode of acute stress prior to commencement of symptoms; moreover, catheterization findings did not show findings of Takotsubo cardiomyopathy. Although the patient was noted to have irregularities of coronary vasculature, the evidence for single-vessel fixed stenosis causing intermittent chest pain could not be established. The patient was maximized on medical therapy secondary to diffuse coronary atherosclerosis; however, it still did not suffice the presentation of ST-elevation myocardial infarction (STEMI) based on EKG findings.

## Discussion

Although few case reports in the past have described coronary vasospasm, with this case report, we have tried to describe one of the rare cases of coronary vasospasm with significant troponin elevation in the setting of transient EKG changes [13]. In another case report, a patient with cannabinoid hyperemesis syndrome was reported to have STEMI with coronary vasospasm, without any troponin elevation, and Echo findings consistent with Takotsubu cardiomyopathy [14]. Additionally, cerebral vasospasms have also been mentioned in a case report of a younger adult with marijuana use [15], most likely due to the disturbance of the vascular tone and compromised blood supply to the brain [16], leading to symptoms mimicking cerebrovascular accident or transient ischemic attack. Based on literature research and our case report, it is evident that coronary vasospasm is more likely to be related to synthetic marijuana use because it is more potent and combined with multiple chemicals and possibly has an association with some biochemical transformation of THC with interaction with another compound present in synthetic marijuana.

The diagnosis of coronary vasospasm is challenging and requires similar evaluations compared to acute coronary syndrome, secondary to initial EKG changes; however, the timeline for cardiac catheterization can be assessed when there is a high suspicion of polysubstance use. In the ED setting, in a patient presenting with chest pain and initial EKG changes including ST changes and T-wave inversion, with significant troponin elevation, it is almost impossible to differentiate vasospastic phenomenon from acute myocardial infarction. Based on the case we have described, there is a role for repeating EKG to reassess whether EKG changes were transient. In EDs where coronary CTs are used, imaging can be obtained after normalization of EKG changes back to baseline. In this scenario, although the patient would eventually require cardiac catheterization, a coronary CT scan would help in risk stratifying whether or not there is a need for urgent cardiac catheterization with STEMI protocol. Another factor in establishing vasospasm and repeating EKG is the administration of intraoperative nitro as it not only improves chest pain but is also a well-established modality for vasospasm in smooth muscle.

## Conclusions

Chest pain associated with cocaine in the past has been a common presentation in the ED setting; however, synthetic marijuana-associated coronary vasospasm represents a significant challenge, with elevated biomarkers and EKG changes, as was the case with our patient, who did not have any other positive substances noted in his urine. There are many impediments in identifying this presentation as information regarding synthetic marijuana is not as well described in the literature as traditional THC, along with the uncertainty of substances that have been combined. In most instances, ED physicians have to rely on patients’ accounts of events because not all rapid urine drug tests utilized in the ED are capable of detecting synthetic marijuana. Although there is always a low threshold for initial cardiology evaluation in these cases, urgent cardiac catheterization should be based on EKG findings, physician’s judgment, and risk stratification in consultation with cardiology along with repeating EKG within minutes after administration of nitro. In the future, we need to further analyze the mechanisms of synthetic marijuana on coronary vasculature to establish a more well-defined framework and parameters to treat these patients with an acute presentation.
